# High glucose enhances fibrosis in human annulus fibrosus cells by activating mTOR, PKCδ, and NF-κB signaling pathways

**DOI:** 10.18632/aging.205876

**Published:** 2024-05-29

**Authors:** Chun Tseng, Shan-Chi Liu, Xiu-Yuan He, Hsien-Te Chen, Pang-Hsuan Hsiao, Yi-Chin Fong, Chih-Hsin Tang

**Affiliations:** 1 Graduate Institute of Biomedical Sciences, China Medical University, Taichung, Taiwan; 2 Department of Orthopedic Surgery, China Medical University Hospital, Taichung, Taiwan; 3 Spine Center, China Medical University Hospital, Taichung, Taiwan; 4 Department of Orthopedic Surgery, China Medical University Beigang Hospital, Yunlin, Taiwan; 5 Institute of Biomedical Sciences, Mackay Medical College, New Taipei City, Taiwan; 6 Department of Pharmacology, School of Medicine, China Medical University, Taichung, Taiwan; 7 Department of Sports Medicine, College of Health Care, China Medical University, Taichung, Taiwan; 8 Chinese Medicine Research Center, China Medical University, Taichung, Taiwan; 9 Department of Medical Laboratory Science and Biotechnology, College of Medical and Health Science, Asia University, Taichung, Taiwan; 10 Department of Medical Research, China Medical University Hsinchu Hospital, Hsinchu, Taiwan

**Keywords:** high glucose, IVDD, fibrosis, NF-κB

## Abstract

Low back pain stands as a significant factor in disability, largely resulting from intervertebral disc degeneration (IVDD). High glucose (HG) levels have been implicated in the pathogenesis of IVDD. However, the detailed mechanism of HG in IVDD is largely unknown. Our clinical results revealed that fibrosis markers such as CTGF, Col1a1, ATF4, and EIF2 are highly expressed in advanced-stage IVDD patients. Stimulation of human annulus fibrosus cells (HAFCs) with HG, but not mannitol, promotes fibrosis protein production. Ingenuity Pathway Analysis in the GSE database found that the mTOR, PKCδ, and NF-κB pathways were significantly changed during IVDD. The mTOR, PKCδ, and NF-κB inhibitors or siRNAs all abolished HG-induced fibrosis protein production. In addition, treatment of HAFCs with HG enhances the activation of mTOR, PKCδ, and NF-κB pathways. Thus, HG facilitates fibrosis in IVDD through mTOR, PKCδ, and NF-κB pathways. These results underscore the critical role of HG as a fibrotic factor in the progression of IVDD.

## INTRODUCTION

Low back pain stands as a significant factor in disability associated with aging, leading to the highest disability-adjusted life years when compared to other health conditions [1, 2]. Intervertebral disc (IVD) degeneration (IVDD) is a complex condition with a multifactorial etiology, encompassing age-related degeneration, genetic predisposition, nutritional and oxygen deficiencies, mechanical overloading, and pathologic changes within the disc itself [3–6]. Treatment modalities for IVDD range from conservative management to innovative biological and engineering approaches. Hence, understanding the molecular mechanisms of IVDD holds the potential to guide the development of novel therapeutic interventions.

The IVD is an avascular structure composed of the nucleus pulposus, annulus fibrosus (AF), and cartilaginous endplates [7]. Degeneration often involves structural failure and biochemical changes within these components [8]. Fibrosis and inflammation are intimately intertwined in the pathogenesis of various degenerative diseases, acting through complex mechanisms that are only partially understood. Inflammatory responses are known to initiate and exacerbate fibrotic processes, with chronic inflammation often resulting in the persistent deposition of extracellular matrix (ECM) and subsequent tissue scarring [9]. This relationship is not unidirectional; fibrotic tissue can further promote inflammatory responses, creating a vicious cycle that contributes to the progression of degenerative diseases [10, 11]. Matrix metalloproteinases (MMPs) play a pivotal role in this interplay, as they regulate both the breakdown of ECM in inflammation and its deposition during fibrosis [12].

Hyperglycemia-induced inflammation and fibrosis are pivotal in the progression of a myriad of diseases, acting through various mechanisms to exacerbate cellular and systemic dysfunction [13, 14]. The interplay between high glucose levels and inflammatory pathways has been shown to aggravate pancreatic inflammation and fibrosis, with studies suggesting that the renin-angiotensin axis activated by hyperglycemia plays a significant role in this process [15]. In the cardiovascular system, hyperglycemia has been implicated in modulating collagen expression and the functional differentiation of cardiac fibroblasts, leading to cardiac fibrosis [16]. The direct relationship between hyperglycemia, oxidative stress, and the inflammatory process is well-established, contributing to the chronicity of diseases [17, 18]. Moreover, hyperglycemia has been recognized to impair tissue healing by promoting a prolonged inflammatory response [19] and to synergize with hypoxia in sustaining a pro-inflammatory state in macrophages [20].

High glucose (HG) levels have been implicated in the pathogenesis of IVDD [21]. However, the detailed mechanism of HG in fibrosis on IVDD is largely unknown. Here, we found that the fibrosis markers are associated with the progression of IVDD. HG enhances fibrosis protein expression in human annulus fibrosus cells (HAFCs). The mTOR, PKCδ, and NF-κB pathways mediate HG-induced fibrosis. This establishes that HG is a critical factor for the development of IVDD.

## RESULTS

### Positive correlation between fibrosis markers and the gradient of IVDD

**Figure 1:**
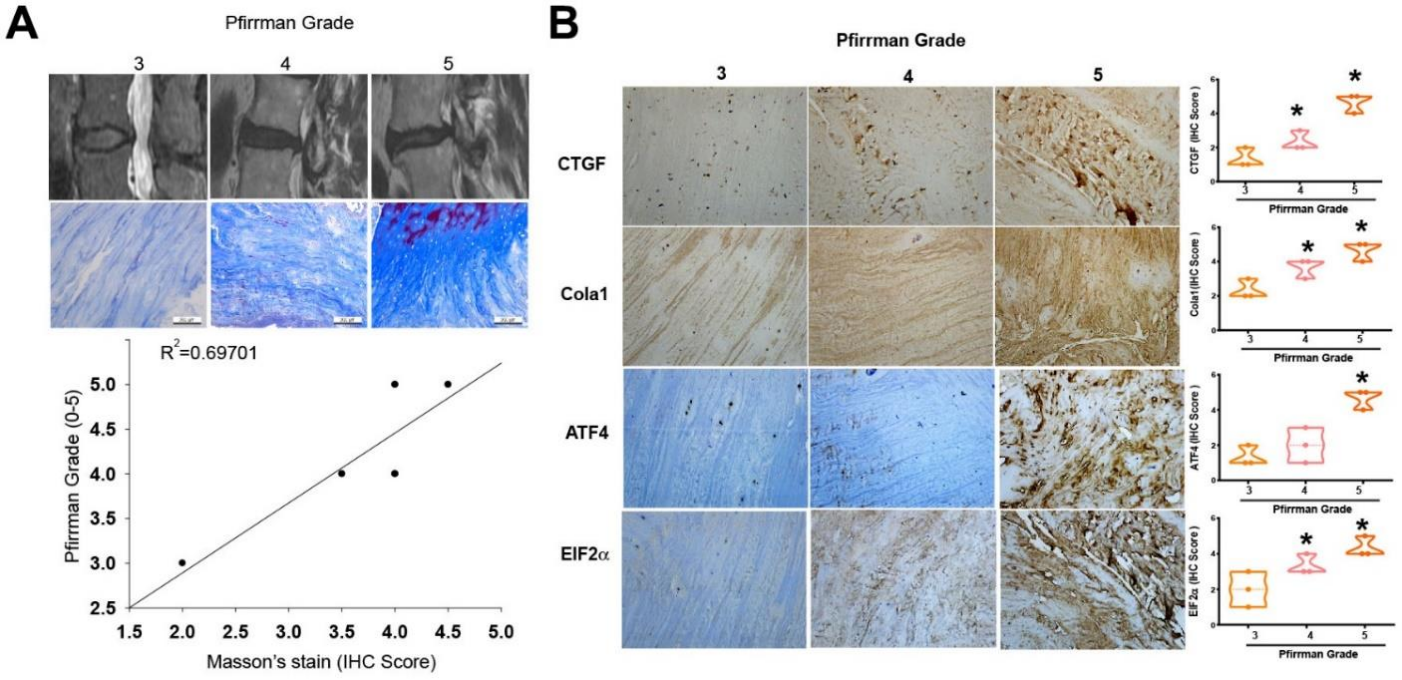
**Higher levels of fibrotic markers in high-grade IVDD patients.** (**A**) MRI images and Masson’s trichrome staining of disc tissues from IVDD patients. (**B**) IHC staining (n=3) was performed for CTGF, Col1a1, ATF4 and EIF2 levels in disc tissues from IVDD patients, followed by photography and quantification. * *p* < 0.05 versus the Grade 3 group.

Fibrosis is a critical process in the development of IVDD [9]. We first investigated the role of fibrosis in the pathogenesis of IVDD. To validate the association between tissue fibrosis and varying grades of disc degradation, we utilized MRI and Masson’s trichrome staining. The results confirmed a positive correlation between tissue fibrosis and Pfirrmann grading (Figure 1A; Spearman R=0.69701). Furthermore, IHC staining was employed to identify the upregulation of fibrotic markers, including CTGF, Collagen Type I (Col1a1), ATF4, and EIF2, in tissues manifesting advanced-stage IVDD procured from clinical specimens (Figure 1B).

### HG enhances expression of fibrotic proteins in HAFCs

**Figure 2:**
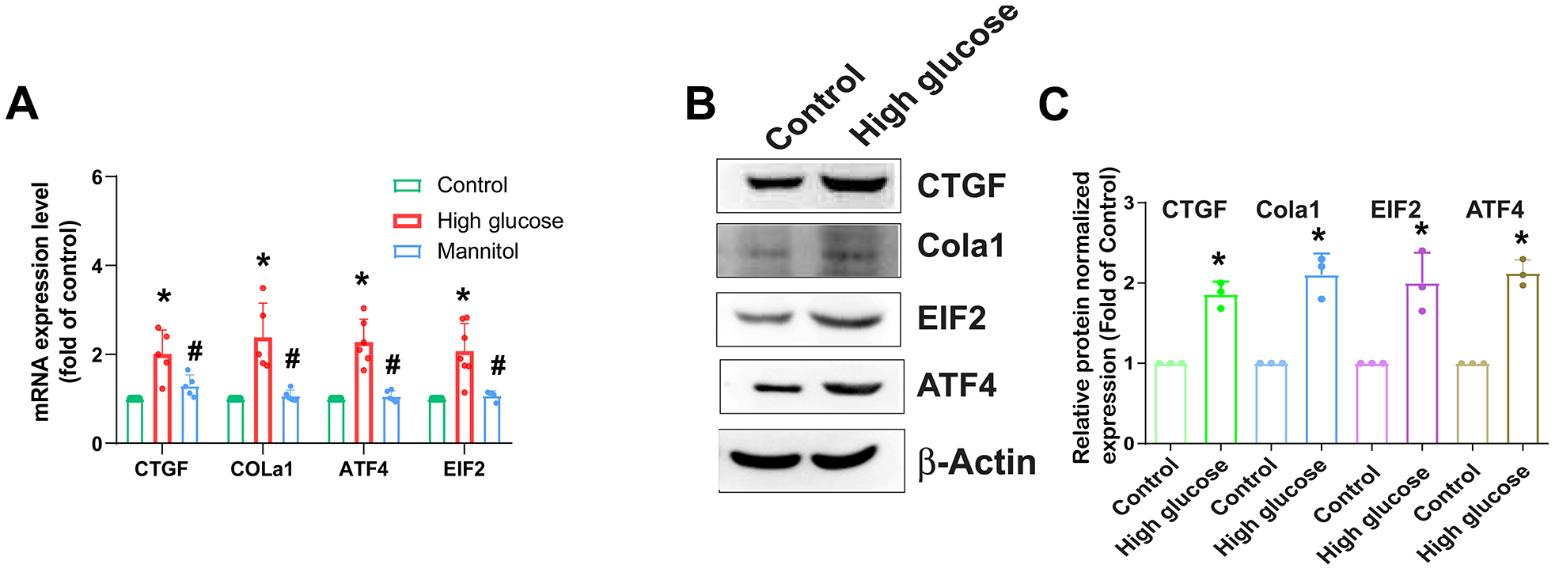
**HG enhances fibrotic protein expression in HAFCs.** HAFCs were treated with glucose (33 mM) or mannitol (33 mM) for 24 h, and the indicated mRNA (**A**) and protein (**B**) expression was examined by qPCR (n=5) and Western blot (n=3). (**C**) The densitometry analysis of (**B**) was quantified. * *p* < 0.05 versus the control group.

AF plays an essential role in the mechanical functionality of the IVD [7]; therefore, HAFCs were used to examine the role of HG in the expression of fibrotic proteins. Stimulation of cells with HG (33 mM) enhances mRNA and protein expression of fibrotic proteins such as CTGF, COL1a1, ATF4, and EIF2 (Figure 2). As osmotic controls, treatment with 33 mM mannitol also did not induce significant changes in the gene and protein levels of CTGF, COL1a1, ATF4, and EIF2 (Figure 2), indicating that the elevated expression of fibrotic proteins induced by HG is not attributable to increased osmolality within the media.

### mTOR and PKCδ signaling pathways are involved in HG-induced increase of fibrosis proteins

**Figure 3:**
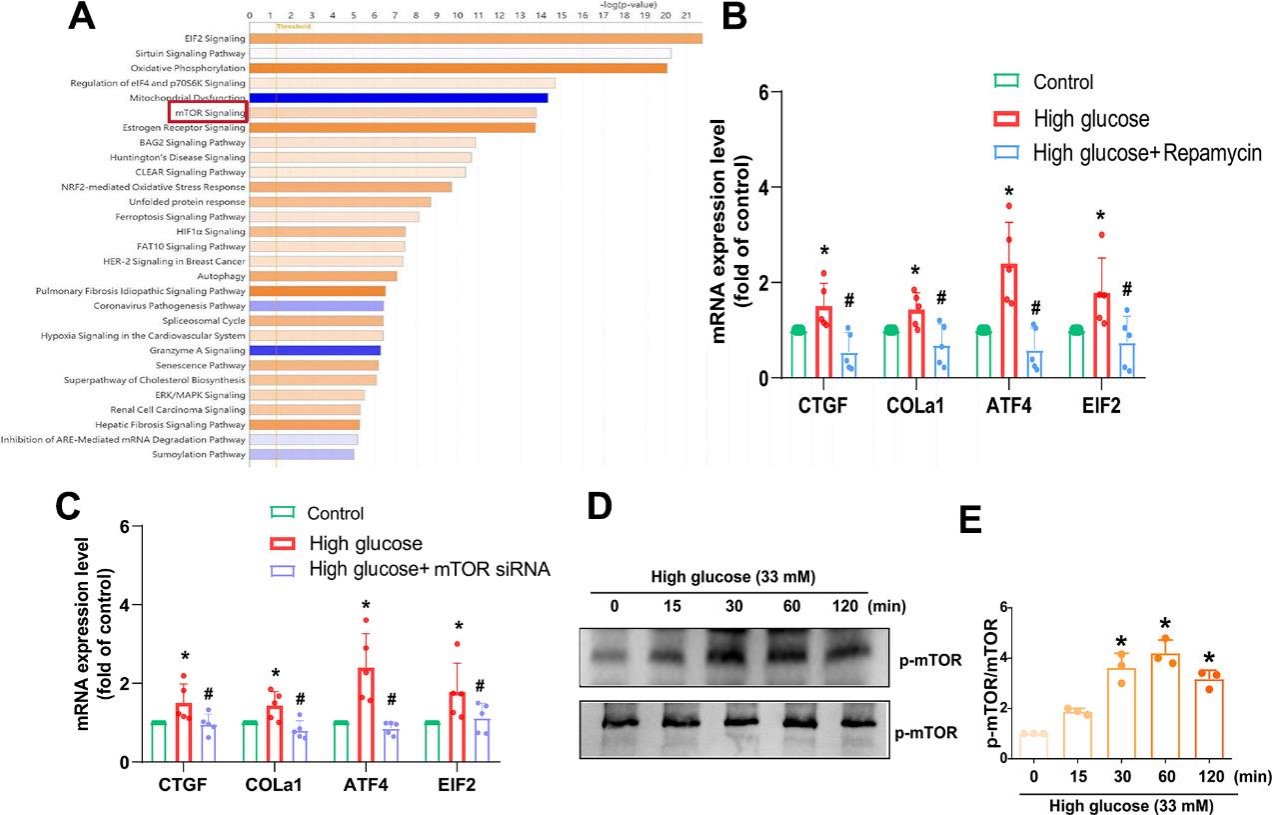
**mTOR is regulated in HG-promoted fibrotic protein expression in HAFCs.** (**A**) IPA pathway enrichment figure showing pathways in the GSE219145 dataset that significantly changed. (**B**, **C**) HAFCs were treated with mTOR inhibitor (rapamycin; 10 μM) or transfected with mTOR siRNA then treated with HG, and the indicated mRNA expression was examined by qPCR (n=5). (**D**) Cells were stimulated with HG, and the p-mTOR expression was examined by Western blot (n=3). (**E**) The densitometry analysis of (**D**) was quantified. * *p* < 0.05 versus the control group. # *p* < 0.05 versus the HG-treated group.

We next sought to examine the regulatory mechanism underlying IVDD by investigating molecular pathways in the GSE219145 dataset using IPA software. Our data revealed a significant correlation between the mTOR, PKCδ, and NF-κB signaling pathways, which is the top signaling (mTOR signaling) in IVDD (Figure 3A). Pretreatment with the mTOR inhibitor (Rapamycin) or transfection with mTOR siRNA reduced HG-induced fibrosis proteins expression (Figure 3B, 3C). Treatment of HAFCs with HG induced time-dependent phosphorylation of mTOR (Figure 3D, 3E). Taken together, it appears that the mTOR signaling pathway regulates HG-induced fibrosis in IVDD.

**Figure 4:**
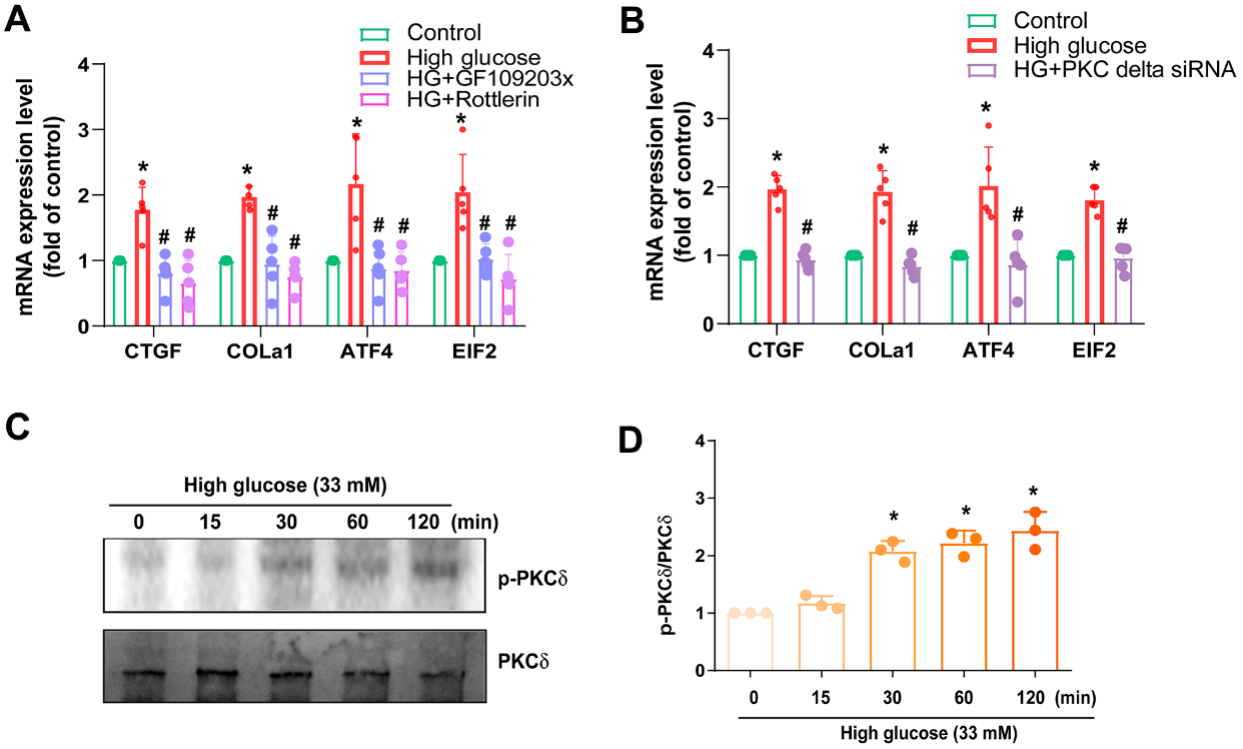
**PKCδ is regulated in HG-promoted fibrotic protein expression in HAFCs.** (**A**, **B**) HAFCs were treated with PKC inhibitor (GF109203x; 10 μM), PKCδ inhibitor (rottlerin; 10 μM) or transfected with PKCδ siRNA then applied with HG, and the indicated mRNA expression was examined by qPCR (n=5). (**C**) Cells were stimulated with HG, and the p-PKCδ expression was examined by Western blot (n=3). (**D**) The densitometry analysis of (**C**) was quantified. * *p* < 0.05 versus the control group. # *p* < 0.05 versus the HG-treated group.

PKC served as a common downstream signaling of the mTOR pathway. Treatment of cells with the PKC inhibitor (GF109203x), PKCδ inhibitor (Rottlerin), or transfection with PKCδ siRNA diminished HG-promoted fibrosis proteins expression (Figure 4A, 4B). Incubation with HG augmented the phosphorylation of PKCδ (Figure 4C, 4D), suggesting that PKCδ activation is controlled in the HG-induced induction of fibrosis in IVDD.

### NF-κB signaling pathway controls HG-induced fibrosis

**Figure 5:**
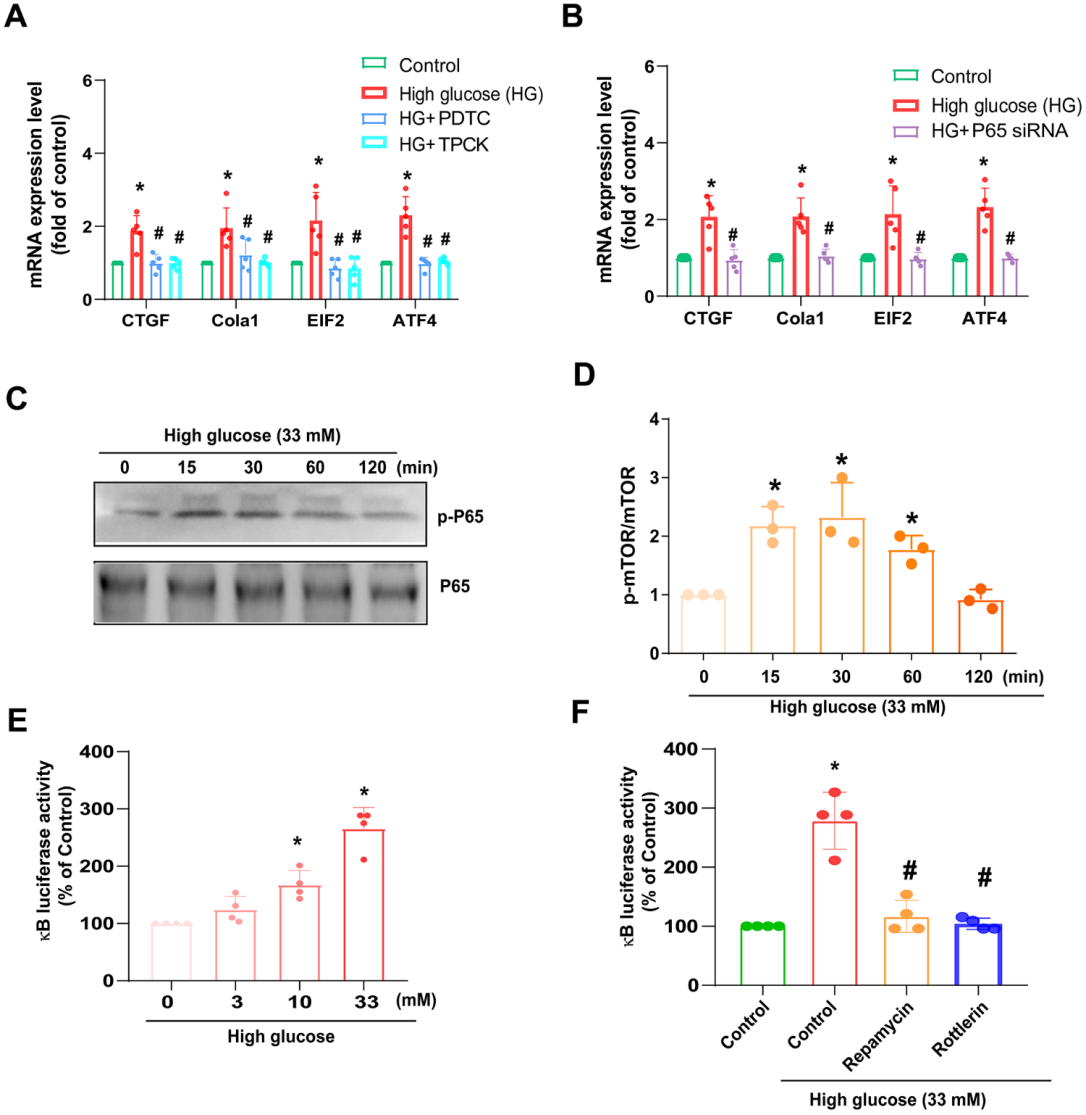
**HG induces NF-κB-mediated fibrotic protein expression in HAFCs through mTOR and PKCδ pathways.** (**A**, **B**) HAFCs were treated with NF-κB inhibitors (PDTC and TPCK; 10 μM) or transfected with p65 siRNA then applied with HG, and the indicated mRNA expression was examined by qPCR (n=5). (**C**) Cells were stimulated with HG, and the p-p65 expression was examined by Western blot (n=3). (**D**) The densitometry analysis of (**C**) was quantified. (**E**, **F**) HAFCs were treated with HG (3 – 33 mM) or pretreated with rapamycin or rottlerin then applied with HG, and the NF-κB luciferase activity was examined (n=5). * *p* < 0.05 versus the control group. # *p* < 0.05 versus the HG-treated group.

NF-κB is a pivotal transcription factor that responds to inflammatory reactions during IVDD [22]. HAFCs were incubated with NF-κB inhibitors, such as PDTC and TPCK; both diminished HG-enhanced fibrosis protein synthesis (Figure 5A). Conversely, siRNA against p65 had similar effects (Figure 5B). Treatment of cells with HG facilitated phosphorylation of p65 (Figure 5C, 5D). We also used NF-κB luciferase activity to further examine the activation of NF-κB [23]. As shown in Figure 5E, HG stimulation of cells resulted in increased NF-κB luciferase activity concentration dependently (Figure 5E). The enhancement of NF-κB activity by HG was reduced by mTOR and PKCδ inhibitors (Figure 5F), indicating NF-κB activation is mediated in HG-promoted fibrosis through mTOR and PKCδ pathways.

## DISCUSSION

IVDD is a prevalent cause of low back pain in middle-aged and older adults, affecting around 40% of the global population. It not only negatively impacts the quality of life for patients but also places a burden on the healthcare system and society at large [24, 25]. Elevated blood glucose levels have been linked to the development of IVDD, with complex underlying mechanisms involving molecular, cellular, and metabolic processes working together to cause fibrosis and disc degeneration [21]. However, the detailed mechanism of HG in fibrosis in IVDD is largely unknown. In our investigation, we found that HG enhances the production of fibrotic proteins in HAFCs. The mTOR, PKCδ, and NF-κB pathways mediate HG’s effects.

A well-known technique for evaluating IVDD that has been linked to clinical symptoms of disc degeneration is the Pfirrmann grading system. It facilitates communication between radiologists and spine surgeons by providing a reliable grading system with good inter- and intra-observer agreement [26]. Research has also shown a relationship between the disc’s fibrosis indicators and the Pfirrmann grading. Strong CTGF expression, for example, may be linked to disc degeneration and fibrosis in painful discs; this suggests that fibrosis markers are, indeed, correlated with alterations detected in the Pfirrmann grading [26]. Here, we found that fibrotic markers such as CTGF, Col1a1, ATF4, and EIF2 are associated with advanced stages of IVDD according to the Pfirrmann grading, indicating that fibrosis is a critical step in the progression of IVDD.

mTOR and PKCδ have been identified as potential candidate signaling molecules mediating HG-regulated cellular responses [27, 28]. Our data obtained from the GSE dataset using IPA software indicated that the mTOR and PKCδ signaling pathways are related with the top signaling pathway (mTOR signaling). In line with this, our investigation revealed that inhibitors targeting mTOR and PKCδ effectively counteracted the HG-induced enhancement of fibrotic protein expression. Additionally, employing genetic inhibition via mTOR and PKCδ siRNAs yielded similar outcomes. Following incubation with HG, HAFCs exhibited increased phosphorylation of mTOR and PKCδ, suggesting the activation of the mTOR and PKCδ signaling pathways by HG in IVDD.

It is well-documented that NF-κB comprises both classical and alternative pathways, mediating a critical role in IVDD progression [22]. HG, known for its potency in enhancing proinflammatory cytokine production, triggers activation of both NF-κB pathways [29]. Indeed, NF-κB pharmacological inhibitors and genetic siRNA were shown to diminish HG-induced fibrotic proteins expression. HG stimulation was also shown to enhance p65 phosphorylation. The mTOR and PKCδ inhibitor abolished HG-induced NF-κB luciferase activity, indicating that HG promotes NF-κB-dependent fibrosis in IVDD through activating mTOR and PKCδ pathways.

**Figure 6:**
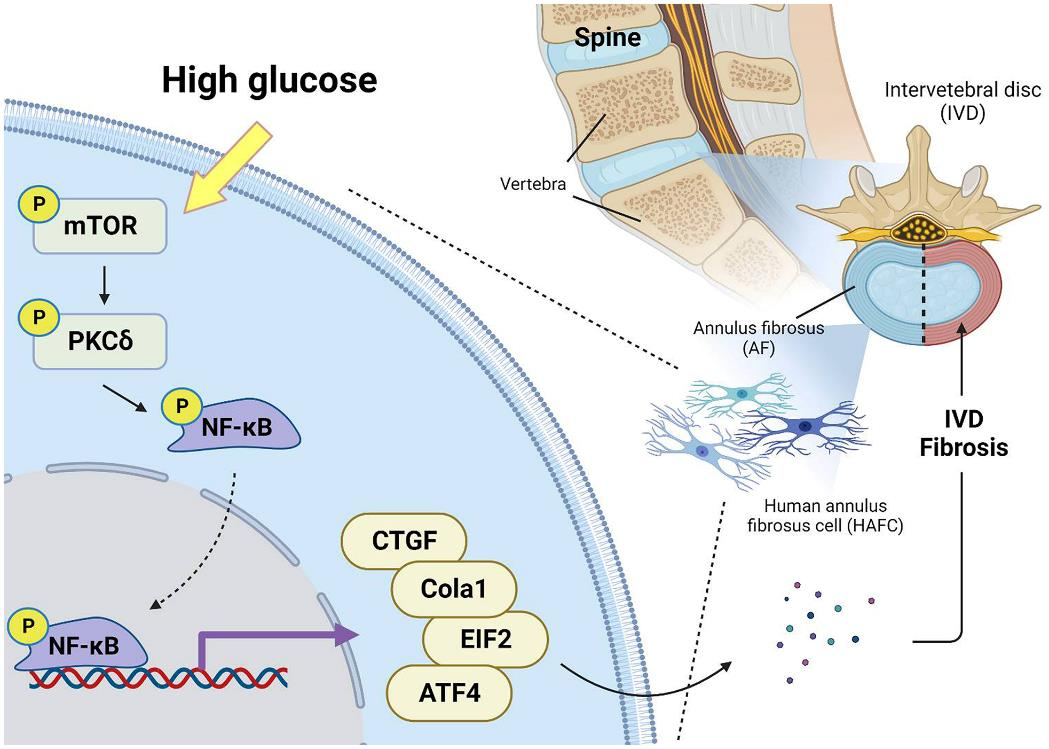
**Schematic illustration showing signaling pathways involved in HG-facilitated fibrosis in IVDD.** HG enhances fibrotic protein expression in HAFCs through mTOR, PKCδ and NF-κB pathways during IVDD progression.

To summarize, the current report indicated that HG promotes the production of fibrotic proteins in HAFCs by activating the mTOR, PKCδ, and NF-κB signaling pathways (Figure 6). These results underscore the critical role of HG as a fibrotic factor in the progression of IVDD.

## MATERIALS AND METHODS

### Material

CTGF (sc-25440), Cola1 (sc-293182), ATF4 (sc-390063) and EIF2 (sc-517627) antibodies were purchased from Santa Cruz Biotechnology (Santa Cruz, CA, USA). All ON-TARGETplus siRNAs were sourced from Dharmacon (Lafayette, CO, USA). Glucose and other chemicals utilized in this study were provided by Sigma-Aldrich (St. Louis, MO, USA).

### Cell culture

HAFCs were purchased from ScienCell Research Laboratories (Walkersville, MD, USA). Cells were cultured in an annulus fibrosus (AF) cell medium. The cells were applied onto culture dishes precoated with 10% Poly-L-Lysine and were incubated in a humidified atmosphere at 37°C with 5% CO_2_.

### Quantitative real-time PCR

qPCR assays were performed using the StepOnePlus sequence detection system in accordance with established protocols [30–32]. Total RNA was isolated from HAFCs using a TRIzol kit (MDBio, Taipei, Taiwan), and an M-MLV Reverse Transcriptase kit (Invitrogen, Carlsbad, CA, USA) was used to perform reverse transcription of total RNA into cDNA. Total cDNA was applied with sequence-specific primers using a KAPA SYBR^®^ FAST qPCR Kit (Applied Biosystems, Foster City, CA, USA) [33, 34]. RT-qPCR assays were carried out in triplicate using a StepOnePlus sequence detection system. The cycling conditions were as follows: an initial 10-minute polymerase activation at 95°C followed by 40 cycles of denaturation at 95°C for 15 seconds and annealing/extension at 60°C for 60 seconds. The threshold was set above the non-template control background and within the linear phase of the target gene amplification to calculate the cycle number at which the transcript was detected (denoted as CT). The primer sequences used were as follows: CTGF forward primer (CAGGCTGGAGAAGCAGAGTCGT) and reverse primer (CTGGTGCAGCCAGAAAGCTCAA); Collagen Type I (Col1a1) forward primer (GAGGGCCAAGACGAAGACATC) and reverse primer (CAGATCACGTCATCGCACAAC); ATF4 forward primer (ATGACCGAAATGAGCTTCCTG) and reverse primer (GCTGGAGAACCCATGAGGT); EIF2 forward primer (TGGTGAATGTCAGATCCATTGC) and reverse primer (TAGAACGGATACGCCTTCTGG); GAPDH forward primer (ACCACAGTCCATGCCATCAC) and reverse primer (TCCACCACCCTGTTGCTGTA).

### Patients and clinical samples

IVD tissues were obtained from 10 patients suffering from lumbar spinal intervertebral disc herniation concurrent with intervertebral degeneration disease. Image studies with completed lumbar spine magnetic resonance imaging (MRI) were all obtained and confirmed the image relative with patients’ symptoms. All patients were scheduled for microdiscectomy with or without interbody fusion surgery. All patients were treated at the China Medical University Beigang Hospital, Yunlin, Taiwan and provided written informed consent prior to participation in the study. All procedures were conducted in accordance with the Institutional Review Board (IRB) regulations and guidelines established by the IRB of China Medical University Hospital, Taichung, Taiwan.

### Western blotting

The proteins from the tested HAFCs were extracted using RIPA buffer. Subsequently, protein samples were electrophoretically separated using SDS-PAGE gels (7.5-12%) and transferred onto PVDF membranes (Merck; Darmstadt, Germany). After blocking with 5% non-fat milk, the membranes were incubated with primary antibodies overnight at 4°C, followed by incubation with specific secondary antibodies for an hour at room temperature. The expression of the target protein was detected using an ECL kit (Millipore, USA) and visualized with the ImageQuant™ LAS 4000 biomolecular imager [35–37].

### Bioinformatics analysis

To determine the underlying pathways involved in the formation of IVDD, we utilized the Ingenuity Pathway Analysis (IPA) to interrogate the GEO database (GSE219145). IPA was used to identify critical pathways related with the significant genes in both regions compared to control. Results from IPA are represented by z-score. Annotations were applied to identify which categories the differentially expressed genes (DEGs) were regulated with key pathways.

### Transient transfection and NF-κB-luciferase assay

ON-TARGETplus siRNAs targeting mTOR (L-003008-00), PKCδ (L-003524-00), and p65 (L-003533-00-0005) were purchased from Dharmacon Research (Lafayette, CO, USA). siRNA (100 nM) was transiently transfected using DharmaFECT1 transfection reagent, according to the manufacturer’s instructions.

HAFCs were cultured in a 6-well plate, and the NF-κB-luciferase plasmid (pNF-κB-Luc; QYB0387; Stratagene, San Diego, CA, USA) was transfected into the cells using Lipofectamine™ 2000. The luciferase activity value was normalized to transfection efficiency, which was monitored by the co-transfected β-galactosidase expression vector.

### Masson’s trichrome staining

IVD tissues were fixed in 4% paraformaldehyde, embedded in paraffin, and serially sectioned at 5-μM thickness. The sections were stained with Masson’s trichrome staining kit (TRM-2; Scytek Laboratories, UT, USA) to measure collagen deposits. Three adjacent sections were quantified using ImageJ software.

### Immunohistochemistry (IHC) staining

Immunohistochemistry assays were conducted on tissue specimens obtained from IVDD patients. The primary antibodies employed in the IHC procedure were CTGF, Cola1, ATF4 and EIF2. The quantification was carried out following the protocol detailed in our prior publications [34, 38]. The IHC staining was assigned scores ranging from 1 to 5 (from weak to strong) to denote positive expression [39].

### Statistics

All statistical analyses were carried out using GraphPad Prism 5.0 (GraphPad Software) and all values are presented as the mean ± standard deviation (SD). Statistical significance between experimental groups was evaluated using the Student’s t-test. For comparisons involving more than two groups, one-way analysis of variance (ANOVA) was employed, followed by Bonferroni’s post hoc test. Differences between groups were considered significant if the *p*-value was < 0.05.
